# Benchmarking Wearable Robots: Challenges and Recommendations From Functional, User Experience, and Methodological Perspectives

**DOI:** 10.3389/frobt.2020.561774

**Published:** 2020-11-13

**Authors:** Diego Torricelli, Carlos Rodriguez-Guerrero, Jan F. Veneman, Simona Crea, Kristin Briem, Bigna Lenggenhager, Philipp Beckerle

**Affiliations:** ^1^Cajal institute, Spanish National Research Council (CSIC), Madrid, Spain; ^2^Robotics and Multibody Mechanics Research Group, Department of Mechanical Engineering, Vrije Universiteit Brussel and Flanders Make, Brussels, Belgium; ^3^Hocoma AG, Volketswil, Switzerland; ^4^The BioRobotics Institute, Scuola Superiore Sant'Anna, Pontedera, Italy; ^5^IRCCS Fondazione Don Carlo Gnocchi, Florence, Italy; ^6^Department of Physical Therapy, Faculty of Medicine, Research Centre of Movement Science, University of Iceland, Reykjavík, Iceland; ^7^Department of Psychology, University of Zurich, Zurich, Switzerland; ^8^Elastic Lightweight Robotics Group, Department of Electrical Engineering and Information Technology, Robotics Research Institute, Technische Universität Dortmund, Dortmund, Germany; ^9^Institute for Mechatronic Systems, Department of Mechanical Engineering, Technical University of Darmstadt, Darmstadt, Germany

**Keywords:** benchmarking, wearable robots, function, user experience, methodology

## Abstract

Wearable robots (WRs) are increasingly moving out of the labs toward real-world applications. In order for WRs to be effectively and widely adopted by end-users, a common benchmarking framework needs to be established. In this article, we outline the perspectives that in our opinion are the main determinants of this endeavor, and exemplify the complex landscape into three areas. The first perspective is related to quantifying the technical performance of the device and the physical impact of the device on the user. The second one refers to the understanding of the user's perceptual, emotional, and cognitive experience of (and with) the technology. The third one proposes a strategic path for a global benchmarking methodology, composed by reproducible experimental procedures representing real-life conditions. We hope that this paper can enable developers, researchers, clinicians and end-users to efficiently identify the most promising directions for validating their technology and drive future research efforts in the short and medium term.

## 1. Introduction

Performance evaluation is becoming an urgent issue in wearable robotics. The community strongly needs reliable and replicable testing methods to verify and compare the performance of the numerous and diverse exoskeletal and prosthetic solutions available (Windrich et al., 2016; Price et al., 2019; Torricelli and Pons, 2019). Without clear and quantitative benchmarks, this rapidly expanding market runs the risk of spreading chaotically, losing sight of real users' needs. This situation is aggravated by the fact that the application domains are now rapidly expanding from the healthcare scenario toward industrial and logistic settings, characterized by a multitude of new functional goals and safety constraints (Gopura et al., 2016; Bogue, 2018). This multifaceted picture calls for a multidimensional approach that can guide not only developers in identifying the most efficient path to market introduction and survival, but also users in identifying the best solution according to their unique abilities, desires, expectations, and needs. Fortunately, the scientific community has already addressed some of these issues in the past two decades: hundreds of studies have explored the biomechanical, physiological, and psychological implications of the interaction between humans and wearable robots (WRs) (Beckerle et al., 2017b, 2019; Pinto-Fernandez and Torricelli, 2020). This has been a multidisciplinary endeavor, which has resulted not only in scientific evidence and better robotic prototypes, but also in a plethora of potentially useful evaluation methods and protocols (Ghillebert et al., 2019; Ármannsdóttir et al., 2020; Davis et al., 2020). If well-organized and appropriately conveyed to the relevant users, a careful selection of these methods can become the foundation of a unified and standardized benchmarking ecosystem for WRs. Different international consortia are now targeting this ambitious goal, such as the COST Action for Wearable Robots^1^, the EUROBENCH project (Torricelli and Pons, 2019), the COVR project (Bessler et al., 2018), and the Exskallerate project^2^, as well as the ASTM-driven Exo Technology Center of Excellence^3^, to mention a few.

**Figure 1:**
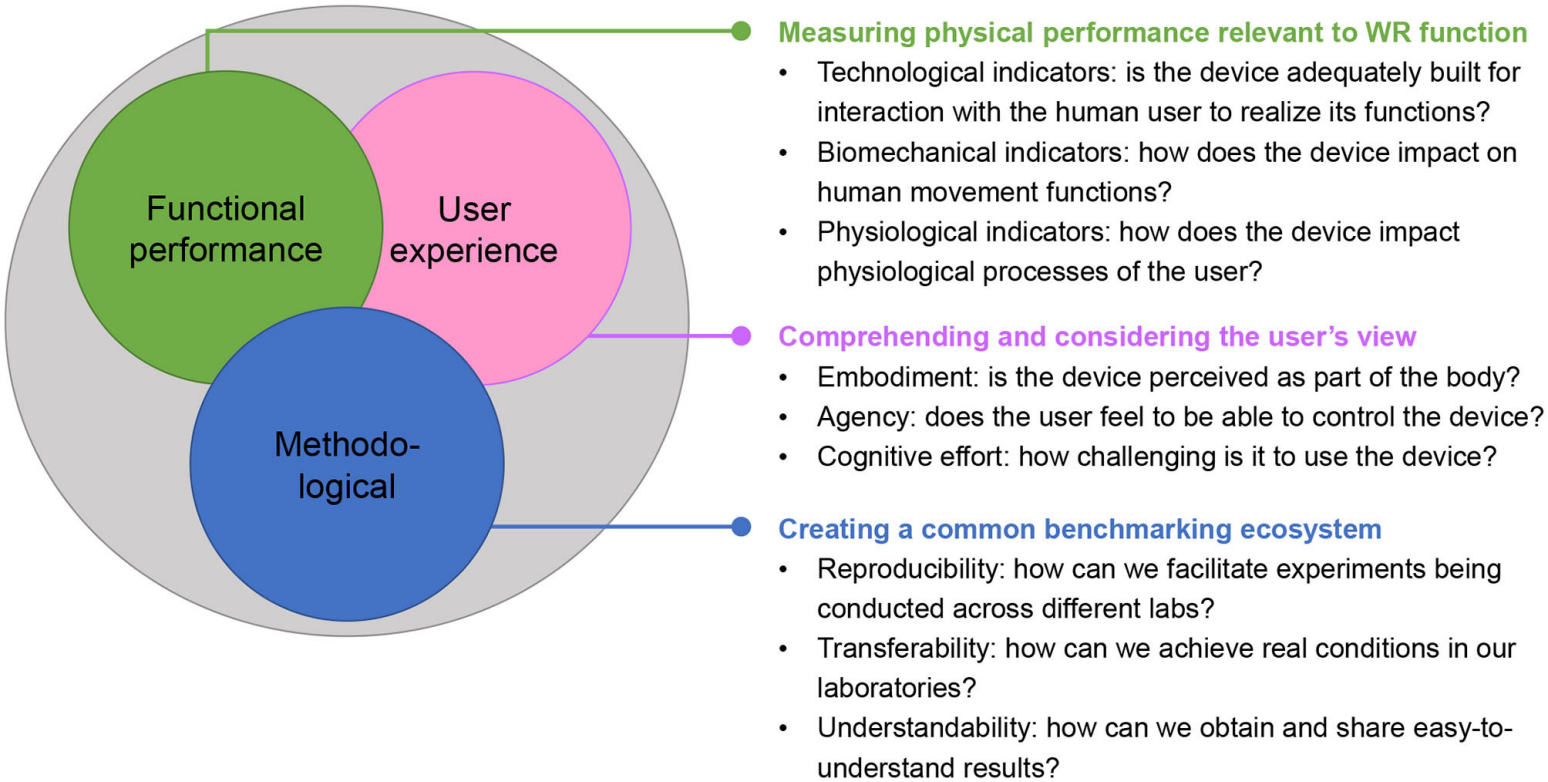
Wearable robot benchmarking should be pushed from several perspectives. We advocate taking a look at functional performance, user experience and methodological aspects. To this end, we consider the sub-aspects and questions to outline research directions.

With the support of some of these projects, we gathered several experts into a workshop titled “Benchmarking Wearable Robots: from key enabling technologies, experimental methods to final applications,” held during the 2019 edition of the ExoBerlin conference^4^. The main goal was to promote the discussion across researchers and stakeholders from different perspectives, to identify the key aspects that should be addressed in the near future in the field of performance evaluation. We identified three areas in which intensive research and scientific discourse appears necessary (see Figure 1). The first one addresses the functional performance, i.e., how the WR interacts with and affects the user's physical functions. Depending on the specific application, performance may be related to different desired outcomes, such as promoting a more physiological and efficient movement pattern, reducing the user's physical fatigue or improving balance. The second one focuses on considering and assessing the user's experience, i.e., the perceptual, emotional, and cognitive processes involved in the use of a WR. The third area highlights the importance of standardizing the experimental procedures, data collection and processing algorithms, in order to ensure a wide adoption of the same testing methods worldwide, fostering discussion and comparison among the different stakeholders in the field.

This perspective paper aims to provide a concise description of each of these three areas and thereby promote a common understanding of the meaning and relevance of WRs benchmarking. Such an effort may enable developers, researchers, clinicians, end-users, and any other relevant stakeholder to focus their efforts toward the most promising directions that should be addressed in the short and medium term.

## 2. Functional Performance Perspective

WRs have an intrinsic, circular causal relationship with the human user, in which the actions of the robot are determinant for the behavior of the human and vice versa. Therefore, performance should be characterized on technological, biomechanical and physiological levels, within the context of specific functional tasks.

Technological indicators describe the physical capabilities of a WR. These indicators are obtained independently from a specific user, but are essential to evaluate the applicability of a WR for a specific target application with human users. One important aspect is the kinematic compatibility, which describes the ability of the robotic structure to follow the 3D kinematic trajectories of the human limbs. Kinematic compatibility is one of the main determinants of relative motion between the human limbs and the device (Näf et al., 2018a), which has direct effects on functionality, comfort and safety. On the kinetic side, the evaluation of torque/force control behavior and the mechanical impedance/admittance characteristics of the actuators is crucial. Unfortunately, most of the existing benchmarking techniques are realized under static assumptions, which result in unrealistic reported actuation characteristics. Recent works are pointing toward dynamic characterization procedures (Moltedo et al., 2019), which in our opinion are essential to measure the potential of a WR to interact safely and efficiently with the human in daily activity tasks.

Biomechanical and physiological indicators relate to the assessment of the physical human-robot interaction. Given the complex nature of the systems under evaluation, i.e., the WR, the user and their physical coupling—the choice of the set of metrics and experimental methods is not trivial. Several biomechanical and physiological metrics have been used in human-in-the-loop studies to assess the effects of a WR on the user's physical capabilities. Among all, kinematics-related metrics have been extensively reported in several works (Pinto-Fernandez and Torricelli, 2020). Comparing joint kinematic profiles in WR-assisted conditions with normative data is a widely used method to assess whether a WR influences the movement pattern of a user (Näf et al., 2018b). In the majority of state-of-the-art papers on lower-limb WRs and gait rehabilitation, gait speed, joint range of motion and spatiotemporal parameters, such as cadence, step width, and stride length, are the most recurring kinematics metrics (Lee et al., 2019). However, given the high diversity of subject conditions, other indicators could be highly relevant to assess the effects of a WR. For instance, assessing the joint torque profiles can provide useful information about the quality of the movement pattern and may guide the interpretation of other outcomes, such as those related to electromyographic (EMG) measurements or metabolic efficiency. EMG measurements have become extremely popular as a way to measure the internal joint dynamics, thus to assess the physiological effects of the human-machine interaction. The technological maturity of commercial systems have made EMG one of the key metrics to evaluate a WR's efficacy in several application scenarios, from rehabilitation, assistance (Collins et al., 2015), and industrial (Pacifico et al., 2020) scenarios, particularly in out-of-the-lab contexts, given that most of the EMG system are wireless and portable. Energy expenditure is currently one of the most adopted metrics to assess the effectiveness of a WR. Reduced metabolic cost has been widely considered as valuable evidence of effective human-robot interaction, with several recent studies proving that such results can be achieved in several contexts, ranging from walking and running (Kim et al., 2019), elderly gait training (Martini et al., 2019), and to repetitive upper-limb assistance of workers (Maurice et al., 2019; Baltrusch et al., 2020; Koopman et al., 2020). Currently, to the author's knowledge, no studies have provided evidence that metabolic cost reductions could be reliably assessed in out-the-lab conditions, but worldwide many research teams are investigating this issue.

Lastly, estimating the interaction forces between the user and the robot is particularly relevant for two main reasons. From a design perspective the assessment of shear and compressive components of the interaction forces can provide useful data to design more comfortable and ergonomic physical interfaces (Langlois et al., 2018), with reduced undesired parasitic forces on the user's musculoskeletal system, wide areas to distribute pressure and tailored coupling with the user's soft tissues. From a functional perspective, the assessment of interaction forces could provide information about the effectiveness and quality of the WR assistance. Despite their great importance, assessing interaction forces may be limited by technological constraints, as either the WR needs to integrate *ad-hoc* force/torque sensors or the experimental set-up should be designed to include sensory systems at the human-robot interface (Donati et al., 2013). Currently, techniques, both accurate and practical, for dynamic in-the-loop pressure measurements are still lacking. Human-machine interaction is one area in which kinetics are of utmost importance. Nevertheless, interaction forces between the user and the machine are likely underestimated and rarely reported in the literature (del Carmen Sanchez-Villamañan et al., 2019).

Considering the complexity and intrinsic variability of measuring human/robot performance indicators for WRs, it is important to further explore the use of models, both software simulations of human robot interaction as well as advanced testing dummies that simulate the human on all relevant aspects. Once such models can be validated for their ability to represent a certain population, and are approved by the community, important gains in efficiency may be reached. Thereby, the wide range of WR application scenarios needs to be taken into account, e.g., medical WRs call for specific biomechanical and/or physiological metrics and exhibit very strict requirements.

## 3. User Experience Perspective

Due to their tight connection with human users, the adequacy of WRs strongly depends on the experience of and the interaction with their users. When assessing the user outcomes of a WR application, experiences will likely reflect the benefits perceived in terms of physical function, but perceptual, emotional, and cognitive aspects also need to be considered. Recent research has explored how to measure, understand, and consider the users' views. For systematic consideration, existing human-oriented design approaches evaluate user experience and integrate it into design processes, e.g., ISO 9241 (Jokela et al., 2003) or human-machine-centered design (Beckerle et al., 2017a). ISO 9241 defines user experience as perception and reactions of a person resulting from the use of a system, i.e., including aesthetic aspects (Hassenzahl and Tractinsky, 2006) or effects of neural plasticity through co-adaptation (Beckerle et al., 2019). Considering user experience early on could help to improve designs already during their development and experience measures could be meshed in the process of co-adaptation. In design, for instance, experience might directly be assessed for particular components, e.g., the intuitivity of a control algorithm, and serve as a predictor of device acceptance and efficacy (Beckerle et al., 2017b), which also relates the users' attitudes and predispositions (Gauthier-Gagnon et al., 1999; Gallagher, 2005; Kammers et al., 2006).

To quantitatively assess and understand users' views and needs in the first place, studies of human factors influencing the experience of the technical system and, in the long term, validated assessment methods are required. To this end, theoretical models of human factors are helpful (Karwowski, 2006; Wilson and Sharples, 2015), but might require customization regarding the specific application: Gauthier-Gagnon et al. (1999), for example, have proposed a model of human factors regarding lower limb prostheses. The model distinguishes between enabling factors, which might be altered by design, as well as predisposing and psychosocial factors. From an engineering point of view, the latter two might appear less important, but on the contrary, the model explains how technical design might not be able to meet a user's needs since unforeseen psychological effects might alter the resulting cybernetic performance, e.g., when the user's perceived security is compromised by the device (Legro et al., 1998; Gallagher and MacLachlan, 2000; Beckerle et al., 2017a). The literature provides extensive information about potentially relevant psychological concepts that influence acceptance and performance of WRs. For some devices, for example, the subjective sense of embodiment (Rognini and Blanke, 2016; Beckerle et al., 2019), the sense of agency over the device (Caspar et al., 2015; Endo et al., 2020), or the subjective cognitive effort (Beckerle et al., 2017a) have been suggested to be crucial.

Human-in-the-loop experiments that get users in touch with prototypal components or system implementations appear promising and may provide useful information about how variations of the technical system modulate the users' experiences (Beckerle et al., 2017b, 2019). Assuming device embodiment, agency, and cognitive effort are promising measures in WR benchmarking: nevertheless, accepted standardized testing procedures are still missing. These might include psychometric tools to evaluate subjective experience (Hart and Staveland, 1988; Longo et al., 2008; Caspar et al., 2015) as well as more objective behavioral measures, e.g., proprioceptive drift for embodiment (Christ and Reiner, 2014), intentional binding techniques for agency (Caspar et al., 2015; Endo et al., 2020), or physiological measures, such as heart rate (Ikehara and Crosby, 2005), electrodermal activity, or neurophysiological measures (Christ and Reiner, 2014). Such systematic measures might not only be used to consider user experience in WR design, but could also be a means to implement adaptive control schemes that coordinate control behavior to improve user experience, e.g., predicting embodiment outcome to foster it by appropriately adjusted control (Schürmann et al., 2019). While physiological measurements and electrical stimulation might support this by exploiting neuroplastic effects, deeper investigation of brain plasticity is subject to ongoing research (McGie et al., 2015; Makin et al., 2017). Future human-machine interfaces might be able to mediate affective signals, and thereby, also forward emotional and social information to the users (Beckerle et al., 2018).

## 4. Methodological Perspective

Turning the existing metrics, protocols, and algorithms into one harmonized benchmarking ecosystem is an important challenge that needs to be addressed for benchmarking to be converted into common practice. This process has to consider several perspectives (see Figure 1) and faces the challenge of finding new and common terminology.

First, benchmarking should allow reproducibility of results, defined as “the obtention of comparable results by different teams, measuring systems, and locations” (Plesser, 2018). The development of a reproducible experiment should clearly consider at least the following four aspects: the physical testbed and environment, the experimental procedure, the data format, and the performance metrics (Torricelli et al., 2015). The concept of reproducibility claims that a range of variations in these elements may not affect the comparability of results, while it greatly improves the chance to be adopted by many users. The main question in this respect is “how different can two testbeds, protocols, measurement systems be to still allow for a truthful comparison?” Currently, there are no guidelines available to help researchers answer this question and to provide a clear description of these components in a standardized way. Fortunately, some editorial initiatives are currently encouraging this direction, e.g., the “R-articles” initiative proposed by (Bonsignorio, 2017). Reproducibility in WRs experiments can be particularly complicated, because the results may be influenced by variables related to human-related aspects that can be hardly controlled or classified, such as the neurological and physical conditions of the user, the amount and type of familiarization with the device, the tuning procedure of the control system, as well as several environmental, i.e., non-technical, factors.

The second aspect is the transferability of results, i.e., the ability of predicting how a system would behave in the real world, by means of experiments conducted in a controlled (typical laboratory) environment. This problem is now becoming more and more relevant due to the increasing number of applications of exoskeletons in diverse contexts. Performing the experiments in a real setting may be either not possible (e.g., in industrial settings) or too complex, due to the multiple variations in the environment, which would imply the execution of an excessive number of experiments. Two promising approaches are the use of complex mechatronic simulators, e.g., the CAREN system from MOTEK, or the decomposition of the complex tasks into basic environmental conditions and motor skills (Torricelli and Pons, 2019). The problem of transferability is particularly complex when it refers to user experience, due to the difficulty to generalize across multiple potential users with variable needs. This may explain why these methods appear to be scarcely applied in the field of WRs up to now (Beckerle et al., 2017b). Moreover, qualitative data can provide very rich information for development processes, but still not very easy to be considered as a hard benchmark.

The third aspect is related to understandability. Benchmarks should not only serve developers and researchers to perform deep technical analysis on their systems, but also to the end-users, to help them compare the different (but apparently similar) solutions available in the market and make the right choice. This can be done only if the user can grasp the main features of the system clearly and quickly. Thus, conveying the benchmarking results using non-technical terminology is of utmost importance. Language should also consider that a single term may have different meanings depending on the user, e.g., medical doctor, industrial stakeholder, generic user, etc., and the related application domain. Last but not least: shareability. Let's consider the hypothetical case in which benchmarking is adopted massively by the WR community worldwide. Where will all those data generated by the different laboratories be stored? Benchmarking, by definition, should allow the comparison with a point of reference. How can such a reference be calculated? How can we derive comparisons? Standards may help in this process by establishing fixed reference values to categorize performance into discrete levels but, in this evolving field, it is more than likely that the performance references will also evolve over time. This calls for a centralized software platform that can gather both data and algorithms, and allow comparisons between the scores obtained by one system with all those already tested. However, there are currently two main barriers that can be identified. First, the availability of researchers and developers to provide access to data obtained on their WRs. In this respect, some questions emerge: at what level of detail need data be shared? To what extent can benchmarking and confidentiality matters coexist? The second potential roadblock is the compliance with privacy regulation, e.g., GDPR, which applies to any experiment generating human sensible data. Overcoming these barriers would considerably increase the probability of benchmarking to be used worldwide, and being converted into the de-facto methodology for evaluation of performance.

## 5. Conclusions

Benchmarking is more than measuring or assessing. It is a methodology that allows the entire innovation chain to be monitored and potentially predicted. Without benchmarks, development efforts risk to reach only a small portion of the market, instead of favoring a global shift of the society toward the inclusion of wearable robotic technologies in daily life. The close interaction between a human and a WR poses special challenges to researchers willing to quantify the different aspects of the symbiotic performance. Several international initiatives are paving the way for a standardized benchmarking ecosystem, which has the ambitious goal of facilitating the matching between user demands and product capabilities.

In this article, we outline the research directions that in our opinion are the main determinants of this endeavor and exemplify the complex landscape into the three main areas here described. In the following, we highlight a number of research questions that, in our opinion, will be key to drive future efforts in the field.

Since functional performance and user experience are in reality highly intertwined to each other, we should ask ourselves: would it be possible to predict the user's view from objective physiological, psychophysiological or biomechanical measurements? If we could do so, this would significantly contribute to speed up testing-development iterations and improve individualizing WRs.

The human and the machine are two intelligent counterparts that should learn to interact with each other to achieve a given goal (Beckerle et al., 2017b, 2019). The particular contributions of both agents to the joint task are not fully understood. Establishing the cause-effect relationship between the internal processes and the achievement of the goals is one of the main challenges in benchmarking research, with tremendous potential benefits. Due to the unavoidable presence of the human in the loop, technology providers may encounter difficulties in demonstrating a certain level of performance for their device. In other words: how can the contribution of the human be excluded when comparing different systems' performances? This problem, clearly evident, e.g., in Cybathlon competition—where the performance strongly relies on the pilot's skills, is an open issue that should be urgently considered (Makin et al., 2017).

Finally, a good measured variable does not mean a useful measure of performance. A typical example is kinematics: having a joint profile closer to human healthy reference, e.g., Winter's data, may not tell anything about stability, efficiency, or safety of the device. Additionally, time profiles are usually difficult to grasp for non-technical users. How can we convert these variables into useful indicators of performance? We advocate that WR research and development should strive for finding the optimal balance between measurable, well-defined, and relatively easy-to-administer benchmarks to improve users' outcomes.

## Author Contributions

DT and PB conceptualized the article and coordinated its development as well as the integration of individual contributions. All authors contributed the content, perspectives, and references as well as discussed and revised the manuscript.

## Conflict of Interest

JV works at Hocoma AG, a company that develops and markets robots for functional movement therapy. SC is a shareholder and scientific advisor of IUVO S.r.l., a spin-off company of Scuola Superiore Sant'Anna, which develops wearable robots. The remaining authors declare that the research was conducted in the absence of any commercial or financial relationships that could be construed as a potential conflict of interest.

## Footnotes

^1^https://www.researchgate.net/project/COST-Action-CA16116-Wearable-Robots-for-Augmentation-Assistance-or-Substitution-of-Human-Motor-Functions

^2^https://northsearegion.eu/exskallerate/

^3^https://www.etcoe.org/

^4^https://www.exo-berlin.de/
